# A Protocol Study to Establish Psychological Outcomes From the Use of Wearables for Health and Fitness Monitoring

**DOI:** 10.3389/fdgth.2021.708159

**Published:** 2021-07-26

**Authors:** Frans Folkvord, Amy van Breugel, Sanneke de Haan, Marcella de Wolf, Marjolein de Boer, Mariek Vanden Abeele

**Affiliations:** ^1^Tilburg School of Humanities and Digital Sciences, Communication and Information Science, Tilburg University, Tilburg, Netherlands; ^2^Open Evidence Research, Barcelona, Spain; ^3^Tilburg School of Humanities and Digital Sciences, Culture Studies, Tilburg University, Tilburg, Netherlands; ^4^imec-mict-UGent, Department of Communication Sciences, Ghent University, Ghent, Belgium

**Keywords:** self-tracking, exercise, body empowerment, sports, psychological outcomes, self-determination theory, psychological effects of health tracking technologies

## Abstract

**Background:** The last few decades people have increasingly started to use technological tools for health and activity monitoring, such as tracking apps and wearables. The main assumption is that these tools are effective in reinforcing self-empowerment because they support better-informed lifestyle decision-making. However, experimental research assessing the effectiveness of the technological tools on such psychological outcomes is limited.

**Methods and Design:** Three studies will be conducted. First, we will perform a systematic review to examine the experimental evidence on the effects of self-tracking apps on psychological outcome measurements. Second, we will conduct a longitudinal field experiment with a between subject design. Participants (*N* = 150) begin a 50-day exercise program, either with or without the aid of the self-tracking app Strava. Among those who use Strava, we vary between those who use all features and those who use a limited set of features. Participants complete questionnaires at baseline, at 10, 25, and 50 days, and provide details on what information has been tracked via the platform. Third, a subset of participants is interviewed to acquire additional qualitative data. The study will provide a rich set of data, enabling triangulation, and contextualization of the findings.

**Discussion:** People increasingly engage in self-tracking whereby they use technological tools for health and activity monitoring, although the effects are still unknown. Considering the mixed results of the existing evidence, it is difficult to draw firm conclusions, showing more research is needed to develop a comprehensive understanding.

**Trial registration:** Netherlands Trial registration: NL9402, received on 20 April 2021; https://www.trialregister.nl/trial/9402.

## Introduction

People increasingly use technological tools for health and activity monitoring (1–4). These activity tracking apps and wearables are considered self-empowering because they can help users make better-informed lifestyle decisions based on their data (5, 6). Recent research, however, suggests that self-tracking technology use may lead to bodily *alienation* rather than empowerment, because it could encourage users to trust technology more than what their own body tells them (7). Because these technologies make explicit all sorts of bodily experiences and processes that would otherwise remain unnoticed (heart rate, burned calories, etcetera), people might develop a more objectified and distant instrumental view on their body as something that needs to be monitored and corrected—at the cost of a tacit, smooth reliance on it (5).

To date, however, there is no empirical research, at least to our knowledge, that examines the mechanisms leading to either bodily empowerment or alienation. This protocol describes the uses of different methodological approaches that we will conduct in a project to investigate under which conditions the use of a self-tracking app fosters bodily empowerment or bodily alienation. More specific, the main objective that this project will study is whether and under which conditions the use of self-tracking technologies empowers or alienates people while doing physical activity.

Collecting health related data with technological tools and platforms is referred to as “self-tracking for health” (5, 7). Self-tracking for health involves a self-monitoring process that relies on the quantification of bodily processes (e.g., heart rate, calories burned) and activities (e.g., step counts, types of sports). People have been self-monitoring their body and life for self-improvement since ancient times (8), although with the introduction of digital technologies, users can (1) collect personal and health data in real-time and at a much larger scale, (2) gain information on parameters that are difficult—if not impossible—to track otherwise, (3) obtain personalized feedback and gamified targets derived from processing of collected data (7), and (4) link their data to other data streams, such as social networking platforms, enabling them to compare with others (4).

There is a widespread belief among technology developers, health professionals and scholars that self-tracking technologies can empower users to make healthier life choices (7–9). For example, following Deci and Ryan's (10). Self-Determination Theory, it is argued that self-tracking technologies fulfill the three needs for empowerment: (1) autonomy, (2) competence, and (3) belongingness. Self-tracking technologies allow users to regulate their physical activity without having to rely on external parties (autonomy), help them reach personal targets and compare their performance with others (competence), and generate belongingness through online endorsements and commenting features (11).

Given their assumed empowering potential, one might expect spectacular usage rates for self-tracking technologies. Recent intervention studies, however, report significant numbers of drop out (11–14), with over 50% of new users dropping out in <2 weeks (6). An explanation for this drop-out might be that some users experience *bodily alienation* rather than bodily empowerment (15). This study adopts a multi-method approach to examine if the use of self-tracking technologies lead to bodily alienation rather than bodily empowerment, and whether these in turn predict future use of self-tracking technology as well as future exercise behavior.

Classical phenomenological theories (16–18) shed light on this possibility. They emphasize that in our normal interactions with the world, our bodies function “transparently”: we are typically not aware of our bodies functioning, such as our hearts beating or our feet moving. Scholars argue that paying conscious attention to these tacit bodily processes may have a disruptive, alienating effect on individuals, because such “hyperreflectivity” (16) disturbs our effortless, smooth flow of acting, like paying attention to your feet disrupts your dancing moves. In reflectively engaging with our bodies, then, we may come to see our bodies as objects or instruments to be tweaked and altered, rather than functioning in the background of our everyday existence.

## Methods and Analysis

**Figure 1:**
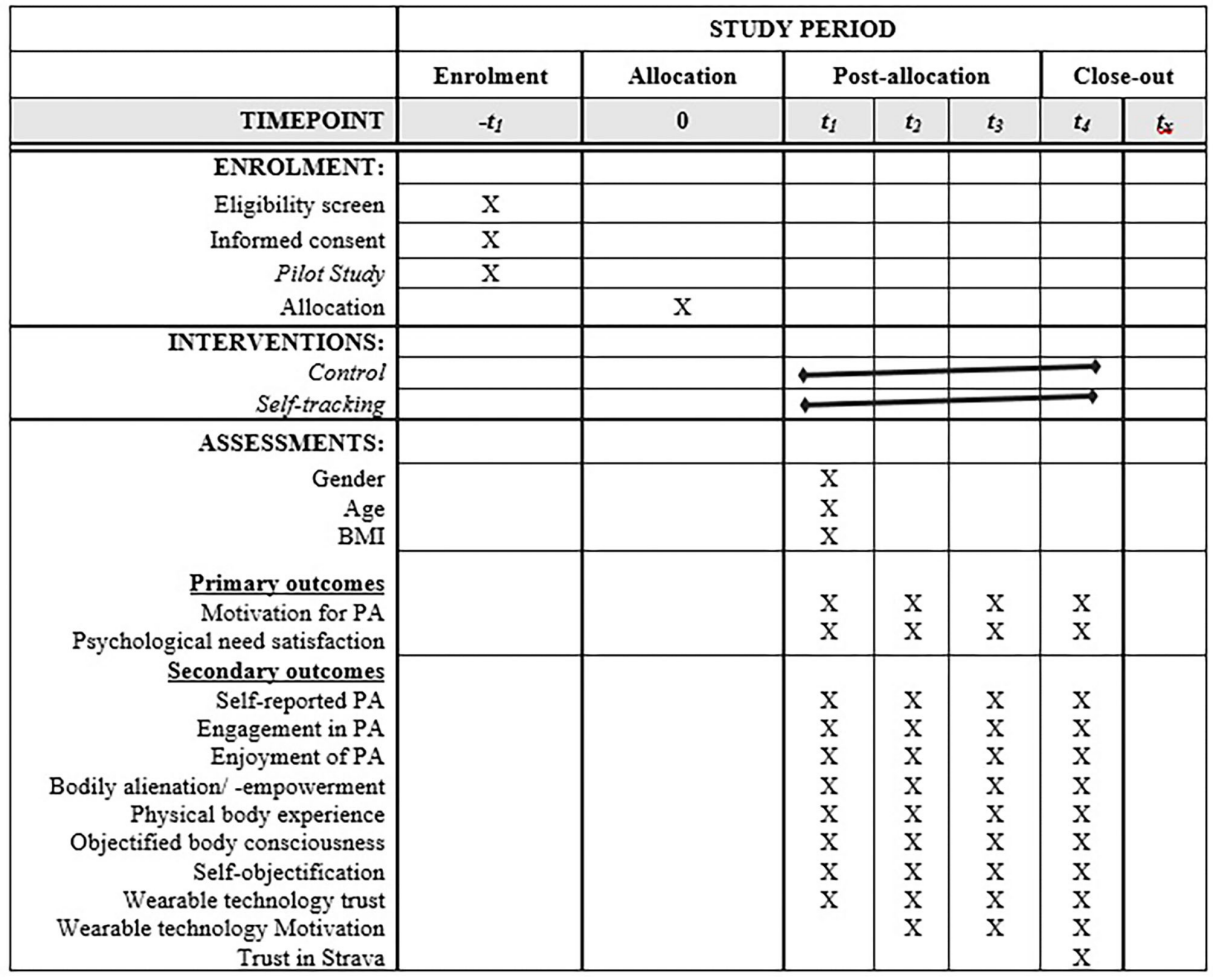
Enrolment, interventions, and assessment in the study period.

In total, three separate studies will be conducted in this project, see for a detailed timeline and phases of the study in Figure 1. First, we will perform a systematic review to examine the experimental evidence on the effects of self-tracking apps on psychological outcome measurements linked to bodily empowerment and alienation to have a state of the art understanding in this area. Second, we will conduct a longitudinal field experiment with a between subject design. Third, a subset of participants is interviewed to acquire additional qualitative data. The study will provide a rich set of data, enabling triangulation, and contextualization of the findings to provide an answer to our research questions. We will provide some more detailed information on the three separate studies here.

### Study 1: Systematic Review

First, to have a state-of-the-art understanding of current existing causal evidence we will conduct a systematic review focusing on experimental studies. As a result of the systematic review we will develop detailed hypotheses that we will test in Study 2: An experimental study and Study 3: Interviews. Participants will be blinded to the hypotheses. We will investigate the following bibliographic databases: PubMed, PsychInfo, and ISI Web of Science, whereby we will restrict our search to peer-reviewed papers or dissertations written in English in the last 10 years (>2010). Next, we will do a manual search in selected articles' reference lists to make sure we do not miss any relevant studies. Studies will be considered eligible if they; (1) report experimental effects of the usage of self-tracking technologies on psychological outcomes, (2) include self-tracking among healthy people and people of any gender and age diagnosed with any type of (chronical) disease, and (3) use self-tracking technologies on one of the following devices for self-tracking; mobile (smart)phones and applications, and/or wearables. Studies will be excluded if they are published before 2010, are not peer-reviewed, are written in a non-English language and have a non-experimental design. The primary outcomes from this systematic review will be used to develop the exact experimental design. For the systematic review, we will follow the PRISMA standard guidelines whereby we provide a detailed explanation to improve transparency. In addition, we will present a flow diagram with all the selection steps of the process and the exclusion criteria.

### Study 2: Experimental Study

Second, we will conduct a longitudinal (50 days) field experiment with a between subject design, aiming for 150 participants that we will collect based on convenience sampling at the Tilburg University campus, see also Recruitment section. We will pre-register our trial (www.trialregister.nl) before we start collecting data and we will include the CONSORT and SPIRIT-statements in our reporting of the outcomes of this study. In this study, we will create two groups that we will randomly allocate to using Strava or not for their physical activity tracking, randomly assigned based on enrolment. An eligibility criterion to participate in the study is that participants must not have used self-tracking technologies for at least 2 months before starting the experiment. Participants complete questionnaires at baseline, after 10 days, 25 days, and a final one after 50 days. We will ask participants to provide details on what information has been tracked via the platform. Next, a subset of participants is interviewed (Study 3).

Power analyses (G^*^Power) (19) with an *F*-test (MANCOVA), suggest that a sample size of *N* = 148 is expected to be sufficient to detect significant (α = 0.05), medium effects (*f* = 0.25) of conditions (power = 0.80). We have based the medium effect size on the study of Seo et al. (20). Considering that some participants might drop out before the last measurement after 50 days, the plan is to include more than 150 participants in the study. We will conduct Multivariate analysis of (Co-)Variance to analyse the primary and secondary outcomes of the experiment.

**Figure 2:**
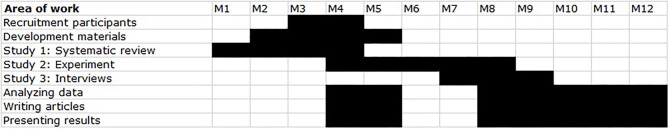
Timetable.

In the experiment we will repeatedly collect different information (see also Figure 2), for example the primary outcomes the motivation to conduct physical activities and psychological need satisfaction, and the secondary outcomes self-reported physical activity, engagement in physical activity, enjoyment of physical activity, experiences of bodily alienation-empowerment, physical body experience, objectified body consciousness, self-objectification, wearable technology trust, wearable technology motivation, and trust in Strava. We will only use validated scales to assess these factors, and will conduct Factor Analysis and Reliability Analyses to make sure the scales we will use are valid and reliable in our dataset as well. All functionalities within Strava, using GPS for recording your physical activities, connecting to people to share recordings of physical activity, comment, or like physical activities of others or see reactions of others on your recordings, share and detect routes, trainings, races, and challenges with your network, connect to a (local) club or community, share photo's, and track and receive feedback on level of fitness and progress, will be part of the study. No specific assessment is considered to be more relevant than other information because the current study will mainly be conducted to test the differences between participants who use a self-tracking app compared to participants who do not use a self-tracking app. In future studies more attention could be given to different functionalities and see the effects of these on psychological or physiological outcomes.

### Study 3: Interviews

Third, we will conduct an in-depth qualitative interview study. For this study, we will invite 10–15 participants to take part in this study. Based on the literature on in-depth qualitative interviewing (19), this number is large enough to allow for saturation of the themes that come up in the interviews while still allowing heterogeneity in findings, also in the event some interviewees will drop out. Recruitment will take place by selecting a subset of participants from the experimental study (study 2) who have volunteered to take part in the interview study. To ensure a heterogeneous sample for our exploratory study, selection criteria include a representation of various gender-identities (the questionnaire provides options for “male,” “female,” and “prefer not to say”) and various levels of user intensity. By using a thematic list for interviewing, including themes such as experiences of health and well-being, exercise routine, and use of the communicative aspect of the Strava app, we provide an overview of the underexplored phenomenon of bodily empowerment and alienation in using Strava, while also providing sufficient focus into bodily experiences of self-trackers.

To analyze the data acquired through this interview study, we will use Interpretative Phenomenological Analysis. This method aims at clarifying how people make sense of their bodies within a larger context (21), and as such, it enables us to identify patterns of bodily alienation and empowerment in the data on using the self-tracking app Strava.

### Recruitment

We will recruit participants at the Tilburg University Campus through online participation platforms for scientific research. Inclusion criterium is that participants should not use a self-tracker already for their physical activities and are between 18 and 35 years old. Participant outside this age range and who already use a self-tracker to record physical activities will be excluded from the study. Participants will receive credits for participating in the study.

### Ethical Approval and Informed Consents

The ethical committee of the Tilburg School of Humanities and Digital Sciences has provided ethical approval for the current study (reference is REDC 2020.198). Explicit written consent to participate will be collected from all participants.

### Confidentiality

Participants will participate in the survey study with a participant number, making them pseudonymous in the survey data, so that they are not directly identifiable in the survey data. There will be a key file (necessary, among others, to assign credits). This key file linking participant number to their name will be stored digitally in a password protected folder. This file will be destroyed once the data have been collected. Identifiable information of the participants participating in the interview study will be pseudonymized when transcribing the data. The recordings made of the interviews will be destroyed immediately after the transcription of each interview. After completion of the study, we will provide all participants with a written debriefing to fully inform them about the study details. We will inform participants to the maximum extent on the usage of the Strava app by providing them with the privacy policy of Strava and asking for them to sign when they have read it and that they comply. In addition, active consent will be collected from all participants.

### Data Management

Considering that a large amount of data will be collected among participants, a specific data management plan and strict protocol has been written in close collaboration with the data steward of the host institute (Tilburg School of Humanities and Digital Sciences, the Netherlands). The host institute is very strict in the treatment of the collected data with the highest level of confidentiality to assure good management of data, which has been tested and evaluated during the ethical approval process.

### Data Monitoring and Auditing

The host institute is very strict in the treatment of the collected data with the highest level of confidentiality to assure good management of data, which has been tested and evaluated during the ethical approval process.

### Risk and Benefits to Participants

We do not consider any risks for the participants in the study. Since we will conduct the study with a student population, the benefits of participating in the study for participants is that they receive credits.

### Knowledge Translation and Dissemination

Considering both the societal and academic relevance of the study, we will proactively communicate and disseminate the outcomes of the proposed studies within this protocol as much as possible. For example, we will aim to publish the outcomes of the study in (open access) scientific international journals and present the results on (inter)national symposiums and conferences. Next, we will seek opportunities to publish the outcomes in non-scientific outlets and present the results at public events as well, in order to disseminate the outcomes of the current project to a wider audience.

## Discussion

This study describes the protocol to systematically test whether and under which conditions the use of self-tracking technologies empowers or alienates, using a multi-method approach. This question thus far remains unexplored.

Digital technologies provide opportunities to the ever more detailed measurement and monitoring of people's activities, bodies, and behaviors in real time. In addition, they offer possibilities for data archiving related to physical activity and for sharing data with peers, directly, or through the social network of users. Because peers can immediately see and react on someone's physical activity that was tracked, the perceived locus of causality, the extent to which individuals perceive their actions as a result of their external or internal reasons, could become externalized. Without using a self-tracker, an individual might be motivated to conduct physical activity because this person likes the activity in itself, while when using a self-tracker, an individual might be motivated to conduct physical activity because of the reactions this person will receive from the personal community afterwards. Via mobile digital devices, for instance, users are continuously connected to the internet, which enables them to immediately inform peers about physical activities and performances. Devices and wearables are typically fitted with digital sensors that assess not only activity and achievements, but also personal and health related information. Such technologies are considered to be a major source of potential revenue for digital developers and entrepreneurs, but empirical evidence showing positive effects remains scarce.

Considering the increasing use and popularity of digital technologies, using these technologies on a day-to-day basis raises important questions concerning their specific psychological effects, and how these may in turn impact future technology use and exercise behavior. Several studies show the effect of self-tracking technologies on endurance (22), well-being and self-determination to conduct physical activity (23), belongingness (20), and sense of competence (23). Bodily empowerment and alienation, however, appear areas that have thus far been largely overlooked.

### Generalizability

Although the proposed experiment and interviews focus on the effects of using Strava, we believe the results of the current study will be generalizable to more self-trackers that have similar functionalities. An increasing number of self-tracker apps and programs are being developed that are able to tracking workouts and provide statistics of these workouts, discover, and connect to local clubs, events and challenges, and interact with personal communities. On the other hand, considering we will mainly focus our study on a student population, we believe the outcomes might be different for older (or younger) target groups because a student population might be more susceptible to social influences than older target groups, or might be more vulnerable to external factors influencing their bodily perceptions.

## Strength and Limitations

One of the strengths of the current study is that until now there have been limited studies examining the effects of using self-trackers on bodily perceptions. To our knowledge, this is the first systematic study that will examine the effects of using self-trackers. Robust scientific evidence is needed, in particular from large-scale Randomized Clinical Trials, with large sample sizes, longitudinal data collection, and predetermined outcome measurements that provide a comprehensive understanding of the usage of self-trackers. Considering the enormous societal success of self-tracking apps, it is essential to improve our understanding and be able to provide evidence-based recommendations about how these technologies influence people, in particular in the long-term.

On the hand, the current study might face some limitation and biases, such as selection bias, given that we will include mostly students that are willing to participate in a study of at least 50 days. Moreover, participants will receive credits for participation in the study, which might be a different motivation than other people who do not receive any rewarding incentive for using the self-tracker. Based on the interviews we will gather motivational aspects of using a self-tracker that we cannot achieve with only an experimental study. In addition, Strava might not be representative for all self-trackers, considering that it is a very popular app with several functionalities that other self-tracking apps do not have.

## Author Contributions

All authors listed have made a substantial, direct and intellectual contribution to the work, and approved it for publication.

## Conflict of Interest

The authors declare that the research was conducted in the absence of any commercial or financial relationships that could be construed as a potential conflict of interest.

## Publisher's Note

All claims expressed in this article are solely those of the authors and do not necessarily represent those of their affiliated organizations, or those of the publisher, the editors and the reviewers. Any product that may be evaluated in this article, or claim that may be made by its manufacturer, is not guaranteed or endorsed by the publisher.
